# A Mutation–Selection Model of Protein Evolution under Persistent Positive Selection

**DOI:** 10.1093/molbev/msab309

**Published:** 2021-10-25

**Authors:** Asif U Tamuri, Mario dos Reis

**Affiliations:** 1 Centre for Advanced Research Computing, University College London, London, United Kingdom; 2 EMBL-EBI, Wellcome Genome Campus, Hinxton, Cambridgeshire, United Kingdom; 3 School of Biological and Behavioural Sciences, Queen Mary University of London, London, United Kingdom

**Keywords:** positive selection, distribution of fitness effects, influenza, RuBisCO, cytochrome b, mutation–selection model

## Abstract

We use first principles of population genetics to model the evolution of proteins under persistent positive selection (PPS). PPS may occur when organisms are subjected to persistent environmental change, during adaptive radiations, or in host–pathogen interactions. Our mutation–selection model indicates protein evolution under PPS is an irreversible Markov process, and thus proteins under PPS show a strongly asymmetrical distribution of selection coefficients among amino acid substitutions. Our model shows the criteria ω>1 (where *ω* is the ratio of nonsynonymous over synonymous codon substitution rates) to detect positive selection is conservative and indeed arbitrary, because in real proteins many mutations are highly deleterious and are removed by selection even at positively selected sites. We use a penalized-likelihood implementation of the PPS model to successfully detect PPS in plant RuBisCO and influenza HA proteins. By directly estimating selection coefficients at protein sites, our inference procedure bypasses the need for using *ω* as a surrogate measure of selection and improves our ability to detect molecular adaptation in proteins.

## Introduction

Understanding how natural selection acts on molecular sequences has long been a pursuit of evolutionary biology. For example, Kimura (1983), using a model that assumes the genome has an infinite number of sites, showed the relative rate of molecular evolution is approximately given by:

(1) $$u=\frac{S}{1-e^{-S}},$$

where *S* is the selection coefficient acting on mutations. If new mutations in the genome are positively selected (*S* > 0) the relative rate of molecular evolution is accelerated (*u* > 1), whereas the rate is the neutral mutation rate (*u* = 1) if there is no selection (*S* = 0), and the rate is decelerated (*u* < 1) if mutations are negatively selected (*S* < 0).

Equation (1), which is the relative probability of fixation of selected over neutral mutations (Fisher 1930; Wright 1931; Kimura 1962; McCandlish et al. 2015), has important implications for understanding molecular adaptation in proteins. For a sample of protein-coding sequences from various species, the ratio between the number of substitutions at nonsynonymous sites (which are under selection) and at synonymous sites (which are under weak or no selection) should approximately follow the dynamics of equation (1) (Nielsen and Yang 2003). This ratio, commonly known as $\omega =d_{N}/d_{S}$, is widely used as a test of molecular adaptation in proteins, with ω>1, *ω* = 1, and ω<1 interpreted as evidence of molecular adaptation (positive selection), neutral evolution, and purifying selection respectively.

However, Kimura’s relative rate of molecular evolution (eq. 1), based on the infinite-sites model (Kimura 1969; Sawyer and Hartl 1992), assumes all new mutations appear at new sites in the genome. This assumption appears unrealistic for proteins. Nielsen and Yang (2003) have argued that if amino acid fitnesses are reassigned every time a new mutation appears at a site in a protein (so that the selection coefficient, *S*, is always the same at the site), then equation (1) gives the relationship of *S* and *ω* under a finite-sites model. However, it is not clear in which condition this fitness reassignment should apply: if an *i* to *j* mutation has selection coefficient *S*, then the reverse *j* to *i* mutation should have coefficient -S, but Nielsen and Yang’s model assumes it reverts to *S*. Without this assumption it does not appear possible to equate $\omega =S/(1-e^{-S})$.

Spielman and Wilke (2015), and dos Reis (2015), used the Fisher–Wright mutation–selection model (Fisher 1930; Wright 1931; Halpern and Bruno 1998) to derive the relationship between *ω* and the selection coefficients acting on codon sites within a finite-sites model. They showed that ω≤1 when selection coefficients are constant over time, that is, they are not reassigned (dos Reis 2015; Spielman and Wilke 2015); whereas ω>1 can be achieved for a short period of time after selection coefficients undergo a single shift during an adaptive event, for example, when a virus adapts to a new host (dos Reis 2015).

However, the relationship between *ω* and selection coefficients under the more general case of persistent changes in selection over time appears unclear. This case, which we term persistent positive selection (PPS), is important because selection coefficients acting at codon sites may change repeatedly during persistent environmental changes, during adaptive radiations, and in host–pathogen interactions (such as in a virus evading herd immunity in a host population). Thus, understanding how PPS affects *ω* in proteins can inform the development of methods to detect positive selection and give us insight onto the mechanisms of adaptive evolution in general.

Here, we develop a mutation–selection model of codon substitution under PPS. The new model can be used to study the mechanistic relationship between the scaled selection coefficients and *ω*, providing insight into the evolutionary dynamics of proteins under PPS. Furthermore, we develop a penalized-likelihood implementation of the model and successfully use it to detect PPS directly in real proteins bypassing the need to use *ω* as a surrogate measure of selection. Analysis under the new model indicates codon substitution is an irreversible Markov process, leading to a highly asymmetrical distribution of selection coefficients among substitutions in proteins under PPS. More strikingly, the PPS model shows the criteria ω>1 to detect molecular adaptation in proteins is conservative and indeed arbitrary, as we find evidence of PPS at codon sites where ω<1.

## New Approaches

### The PPS Codon Substitution Model

We develop the new model by integrating the nonhomogeneous selection model of Kimura and Ota (1970) with the mutation–selection codon substitution model of Halpern and Bruno (1998). Consider a population of organisms with haploid genome number *N*. That is, the number of copies of the genome in the population is *N* (i.e., the population size is *N* if the organism is haploid and N/2 if it is diploid). Suppose a site *k* in a protein-coding gene is fixed for codon *i* in the population, and the scaled Malthusian fitness of *i* is $F_{i,k}$. A new mutant codon *j* appears at the site and has initial selective advantage $S_{ij,k}^{*}=F_{j,k}^{*}-F_{i,k},\;F_{j,k}^{*}>F_{i,k}$. The selective advantage then decays exponentially as a function of time (Kimura and Ota 1970), for example, due to gradual environmental change. Kimura and Ota (1970) showed the fixation probability of *j* is approximately $S_{ij,k}/(1-e^{-S_{ij,k}})\times N^{-1}$ where $S_{ij,k}$ is constant and $0<S_{ij,k}<S_{ij,k}^{*}$. In other words, the fixation probability of *j* is the same as that of an allele with intermediate, but constant, selective advantage $S_{ij,k}$.

It appears other types of decay function lead to the same fixation probability. For example, the same result is obtained in the case of frequency-dependent selection when the fitness of *j* decays exponentially as a function of the frequency of *j* in the population (dos Reis 2013). In the case of frequency-dependent selection, once *j* becomes fixed, any new mutant alleles may have high fitnesses because they would be rare. We expect this type of dynamics in, for example, viruses escaping the herd immunity of a host population. Similarly, if the environment gradually shifts between two states, then the selective advantage of *j* or *i* would be continuously reset depending on the particular environment. This would then lead to resetting (or reassignment) of the fitnesses of *i* and *j*. This persistent change in the selection coefficient is what we term PPS. We formalize codon substitution under the PPS model next.

Let the selection coefficient for the i→j mutation be $S_{ij,k}=F_{j,k}-F_{i,k}+Z_{k}$, where $F_{j,k},\;F_{i,k}$ and $Z_{k}(\geq 0)$ are constant. Let the selection coefficient for the reverse mutant, j→i, be $S_{ji,k}=F_{i,k}-F_{j,k}+Z_{k}$. In other words, we have partitioned the fitnesses of *j* and *i* into two components: a constant component, $F_{j,k}$ and $F_{i,k}$, representing structural constrains of the protein on the amino acid encoded by the codon; and *Z_k_*, the PPS component. Thus, when $Z_{k}>0$, the selection coefficient is persistently reset with new mutations.

The substitution rate from *i* to *j* at location *k*, $q_{ij,k}$, is equal to the neutral mutation rate, *μ_ij_*, times the number of *i* alleles in the population, *N*, times the fixation probability of the *j* mutant (Kimura 1983; Halpern and Bruno 1998). Assuming synonymous substitutions are neutral, this gives:

(2) $$q_{ij,k}=\left\{ \begin{array}{cc} \mu_{ij}\frac{S_{ij,k}}{1-e^{-S_{ij,k}}}, & \text{if}\;\text{the}\;\text{substitution}\;\text{is}\;\text{nonsynonymous},\\ \mu_{ij} & \text{otherwise}. \end{array}\right.$$

### Irreversibility of Codon Substitution under PPS

Equation (2) describes codon substitution as a continuous Markov process. Polymorphisms are ignored and the population is assumed to switch from *i* to *j* instantaneously. This assumption appears reasonable if $N\mu_{ij}\ll 1$, for all *μ_ij_* (Bulmer 1991). The proportion of time location *k* remains fixed for *j* (i.e., the stationary frequency of *j*) is $\pi_{j,k}$. A Markov process is said to be reversible in equilibrium if it satisfies the detailed-balance condition $\pi_{i,k}q_{ij,k}=\pi_{j,k}q_{ji,k}$ (Grimmet and Stirzaker 2004). When *Z_k_* = 0, the model of equation (2) is reversible (Yang and Nielsen 2008). However, when $Z_{k}>0$ the process is, in general, irreversible because the detailed balance condition does not hold. When $Z_{k}>0$, the stationary frequencies are found by solving the system of equations $\sum_{j}\pi_{j,k}q_{ji,k}-\sum_{j}\pi_{i,k}q_{ij,k}=0$ with the constraint $\sum_{i}\pi_{i,k}=1$. We calculate the irreversibility index for site *k* as $I_{k}=|\pi_{i,k}q_{ij,k}-\pi_{j,k}q_{ji,k}|$, where $I_{k}>0$ indicates evolution at site *k* is irreversible, and *I_k_* = 0 otherwise (Huelsenbeck et al. 2002).

### Identifying Protein Locations under PPS

Given an alignment of protein-coding genes with corresponding phylogeny, the model of equation (2) can be used to estimate the $F_{i,k}$ and *Z_k_* using maximum penalized likelihood. To estimate the $F_{i,k}$, we use the Dirichlet-based penalty of Tamuri et al. (2014) and for *Z_k_*, we use an exponential penalty with parameter *λ* (see Materials and Methods). For each site in the alignment, we compare the null model *Z_k_* = 0 (no PPS) against $Z_{k}>0$ (PPS) using a likelihood-ratio test. Because of the boundary condition ($Z_{k}>0$) in the test and the use of penalized likelihood, the distribution of the likelihood-ratio statistic does not follow the typical $\chi^{2}$ distribution. Thus, we use Cox (1962) simulation approach as used in phylogenetics (Goldman 1993) to obtain the appropriate null distribution (see Materials and Methods).

### The Relationship between Selection Coefficients and *ω*

The average substitution rate of codon site *k*, averaged over time is:

$$\rho_{k}=\sum_{i\neq j}\pi_{i,k}q_{ij,k}.$$

This rate can be separated into its nonsynonymous and synonymous components, $\rho_{k}=\rho_{N,k}+\rho_{S,k}$, where,

$$\rho_{N,k}=\sum_{i\neq j}\pi_{i,k}q_{ij,k}I_{N}\;\text{and}\;\rho_{S,k}=\sum_{i\neq j}\pi_{i,k}q_{ij,k}(1-I_{N}),$$

and where the indicator function $I_{N}=1$ if the *i* to *j* substitution is nonsynonymous, and =0 otherwise. For a neutrally evolving sequence (e.g., a pseudogene) the corresponding rates are:

$$\rho_{N}^{\left(0\right)}=\sum_{i\neq j}\pi_{i}^{\left(0\right)}\mu_{ij}I_{N}\;\text{and}\;\rho_{S}^{\left(0\right)}=\sum_{i\neq j}\pi_{i}^{\left(0\right)}\mu_{ij}(1-I_{N}),$$

where $\pi_{i}^{\left(0\right)}$ is the stationary frequency of *i* without selection, which is the same for all sites. Thus, the relative nonsynonymous rate is:

(3) $$\omega_{k}=\frac{\rho_{N,k}}{\rho_{N}^{\left(0\right)}}.$$

See dos Reis (2015) for the full derivation. Spielman and Wilke (2015) give a slightly different definition of *ω_k_* (see also Jones et al. 2016, Youssef et al. 2020).

We note the PPS model is general and has other models as special cases. For example, when $Z_{k}\neq 0$ and $F_{i,k}=F_{j,k}$ for all *i*, *j*, we have:

$$\omega_{k}=\frac{Z_{k}}{1-e^{-Z_{k}}},$$

and the model of equation (2) can be written as $q_{ij,k}=\mu_{ij}\omega_{k}$ if the substitution is nonsynonymous and $q_{ij,k}=\mu_{ij}$ otherwise. In other words, the classic codon models (Muse and Gaut 1994; Yang and Nielsen 1998) are a special case of equation (2) when all codons are assumed to have the same fitness. On the other hand, when *Z_k_* = 0 and $F_{i,k}\neq F_{j,k}$, the model of equation (2) reduces to the mutation–selection model of Halpern and Bruno (1998).

## Results

### Detection of PPS in Simulated Data

Extensive simulations on the estimation of $F_{i,k}$ are available in Tamuri et al. (2012, 2014). Here, our focus is on using simulations to assert whether sites under PPS ($Z_{k}>0$) can be identified using Cox’s method. We simulate codon alignments (1,000 codons in length) on a 512-taxa phylogeny, under various strengths of PPS, with $Z_{k}=0,2,5,$ and 10. The values of $F_{i,k}$ are drawn from random distributions to produce sharp amino acid profiles as in real proteins (see Materials and Methods). These $F_{i,k}$ and *Z_k_* values result in *ω_k_* values roughly between 0.05 and 6 (eq. 3). When *Z_k_* = 0, 6.6% of sites are incorrectly detected to be under PPS, which is slightly higher than the 5% error I threshold (table 1). When the selective advantage is slight ($Z_{k}=2)$, the method roughly identifies 44% of sites under PPS (table 1). The power of the method is excellent and roughly over 95% when the selective advantage is strong ($Z_{k}\geq 5$). We note the exponential penalty on *Z_k_* has a noticeable, albeit slight, effect on the power of the test. When the penalty parameter, *λ* is small, the resulting penalty is diffuse and the penalty is weak. However, as *λ* increases, the penalty becomes stronger with probability density in the exponential moving toward zero. In this case, estimates of $Z_{k}\gg 0$ are more strongly penalized and this translates in a small reduction in the power of the test (table 1). We note the penalized likelihood method used here is essentially the same as posterior mode finding giving our penalties are proper probability densities (Cox and O’Sullivan 1990; Tamuri et al. 2014), and thus the penalties on *Z_k_* and $F_{i,k}$ act as prior densities which regularize the parameter estimates (Cox and O’Sullivan 1990).

**Table 1.** Performance of the LRT for Detecting PPS Sites in Simulated Data after FDR Correction (5%).

True Model	λ=0.01	λ=0.5	λ=1.0
swMutSel	FPR at 0.05 Significance		
(Z=0)	0.066	0.066	0.066
swMutSel+PPS	TPR at 0.05 Significance		
(*Z* = 2)	0.441	0.452	0.449
(*Z* = 5)	0.952	0.952	0.947
(*Z* = 10)	0.965	0.963	0.960

Note.—FPR, false-positive rate; TPR, true-positive rate.

### Detection of PPS in Real Proteins

**Fig. 1.**
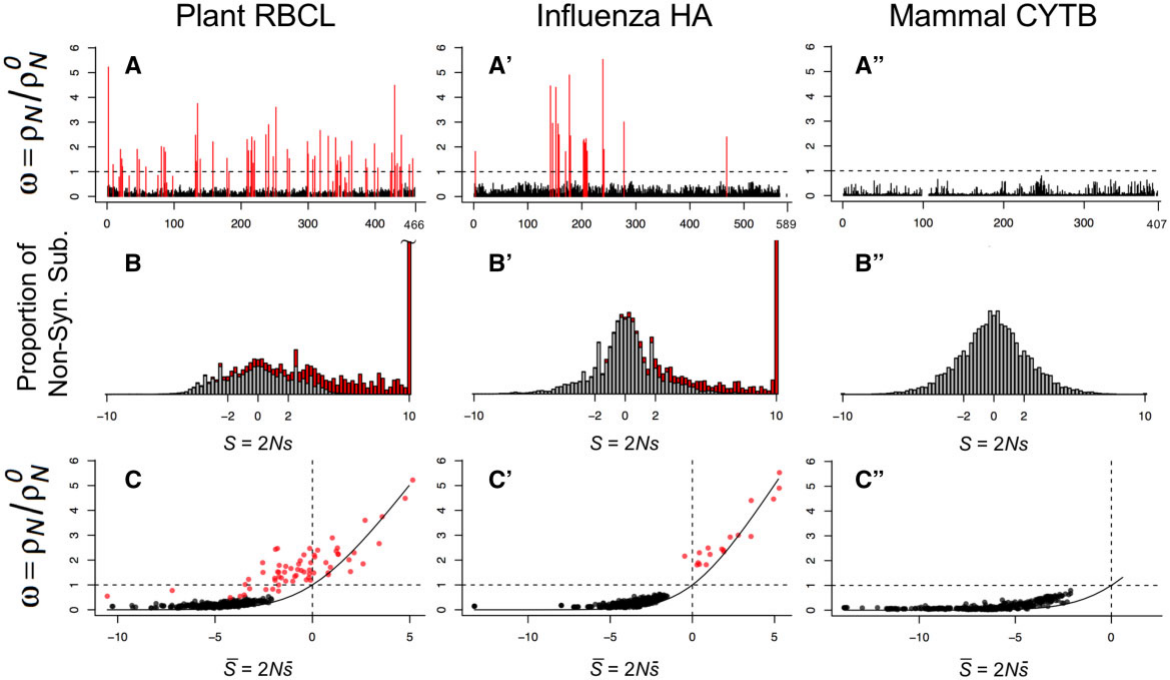
Analysis of proteins under the PPS mutation–selection model. (*A*–*A*″) Estimates of *ω* at protein sites. (*B*–*B*″) Distribution of selection coefficients among nonsynonymous substitutions. (*C*–*C*″) Relationship between *ω* and average selection $\left(\bar{S}\right)$ at protein sites. Sites under PPS ($Z_{k}>0$) are indicated in red in *A*–*A*″ and *C*–*C*″, and their contribution to the distribution of selection coefficients indicated in red in *B*–*B*″. In *C*–*C*′, the solid line is equation (1). The penalty on *Z_k_* is λ=0.001.

We tested for PPS sites in three real sequence data sets: the hemagglutinin protein (HA) from human influenza H1N1 virus, the rbcL protein subunit from flowering plants, and the mitochondrial cytochrome b (CYTB) protein from mammals (table 2). Given the multiple sequence alignment, phylogeny, and mutational parameters, we estimated the $F_{i,k}$ and *Z_k_* using two penalty strengths, λ=0.001, and 0.05. We then performed the LRT of PPS versus no PPS and used false discovery rate (FDR) at the 5% level to identify sites under PPS. Using the weak penalty, λ=0.001, we detected PPS ($Z_{k}>0$) at 65 sites in the plant rbcL and 18 sites in the influenza HA, but we found no PPS sites in mammal CYTB (table 2). Interestingly, only 55 out 65 of PPS sites in rbcL have ω>1. For HA, all 18 PPS sites also have ω>1. The location of PPS sites and estimated *ω_k_* values are shown in figure 1*A* to *A*″. When using the stronger penalty, λ=0.05, the number of sites detected in rbcL and HA are reduced to 50 and 17 sites respectively (table 2). This is not unexpected because, as noted above, stronger penalties push estimates of *Z_k_* toward zero affecting the likelihood ratio test.

**Table 2.** Number of Sites Estimated to be under PPS in Three Real Data Sets.

Data Set	# Taxa	# Sites	# *Z* > 0	(ω>1)
			λ=0.001	λ=0.05
Plant rbcL	478	466	65 (55)	50 (40)
Influenza HA	466	589	18 (18)	17 (14)
Mammal CYTB	418	407	0 (0)	—

### The Distribution of Selection Coefficients at Sites under PPS Is Asymmetrical

**Fig. 2.**
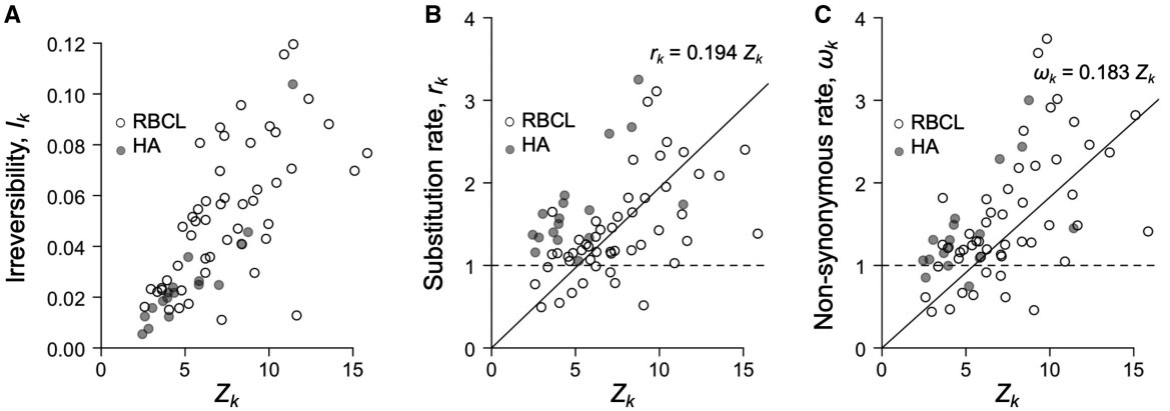
Relationship between *Z_k_* and evolutionary parameters for PPS sites in HA and rbcL. (*A*) Irreversibility index, *I_k_*, versus *Z_k_*. The index is normalized to give the expected excess number of substitutions from detailed balance. (*B*) Site substitution rate, $r_{k}=-\sum_{i}\pi_{k}q_{ii,k}$, versus *Z_k_*. Note the $q_{ij,k}$ are scaled so that they give the relative rate with respect to a neutral sequence (Tamuri et al. 2014). Thus, if *r_k_* = 1, then the site evolves at the same rate as, say, a pseudogene. (*C*) Nonsynonymous rate, *ω_k_* versus *Z_k_*. The penalty on *Z_k_* is λ=0.001 in all cases.

We estimated the distribution of selection coefficients among nonsynonymous substitutions (Tamuri et al. 2014) in the three protein-coding genes analyzed (fig. 1*B* to *B*″). For non-PPS sites (i.e., sites where *Z_k_* = 0), the distribution of selection coefficients is symmetrical, with a mode at *S* = 0, because in this case codon substitution is reversible and the detailed balance condition guarantees the proportions of slightly advantageous and deleterious mutations fixed in the population will be equal over time (Yang and Nielsen 2008). However, among PPS sites in plant rbcL and influenza HA, the distribution is highly skewed with a mode at *S* > 10 because irreversibility of the substitution process means the detailed balance condition does not apply, and hence there is a persistent excess of advantageous mutations being substituted into the population. For example, for sites with $Z_{k}\geq 10$, the irreversibility index is as high as 0.12, indicating there is a deviation of 12% of substitutions from detailed balance, which is a strong deviation (fig. 2*A*). Larger values of *Z_k_* are also associated with faster substitution rates (fig. 2*B*) and larger *ω_k_* values (fig. 2*C*). For example, for sites with $Z_{k}\geq 10$, the corresponding *ω_k_* values range from about 1 to 4 (fig. 2*C*).

### PPS Sites Are under Strong Purifying Constraints

At equilibrium, the average selection coefficient of new mutations at site *k* is:

$${\bar{S}}_{k}=\sum_{i\neq j}\pi_{i,k}P_{ij}S_{ij,k},$$

where $P_{ij}=\mu_{ij}/\sum_{j}\mu_{ij}$ is the probability that the next mutation is *j* given the site is currently fixed for *i* (dos Reis 2015). If most new mutations are very deleterious, then the site is under purifying selection and ${\bar{S}}_{k}<0$; whereas if most new mutations are advantageous, the site is under positive selection and ${\bar{S}}_{k}>0$. Historically, *ω_k_* has been used as a proxy for ${\bar{S}}_{k}$, based on the approximation of equation (1) (Nielsen and Yang 2003). Thus calculating ${\bar{S}}_{k}$ should provide insight into the relationship between the strength of selection at a site and *ω_k_*.

**Fig. 3.**
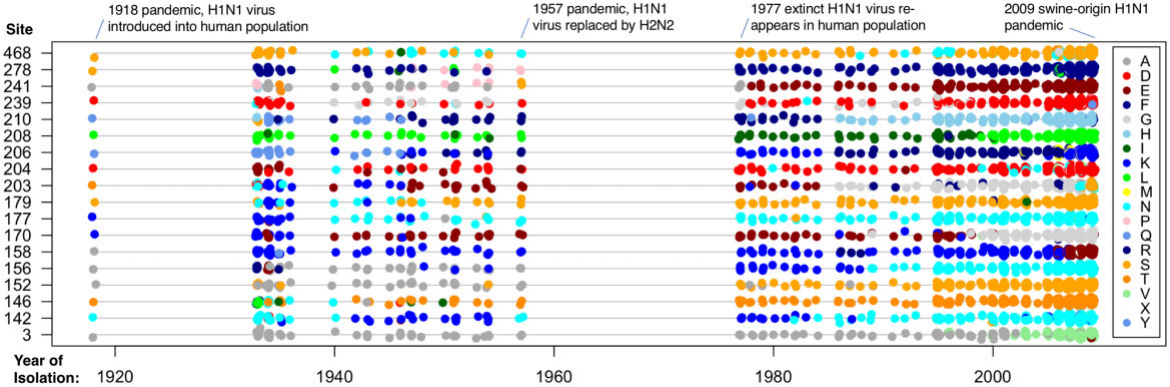
Pattern of amino acid substitution in PPS sites of human influenza (H1N1) HA protein between 1918 and 2009. The penalty on *Z_k_* is λ=0.001. Each colored dot represents a particular amino acid as indicated in the legend.

Figure 1*C* to *C*″ shows the estimated ${\bar{S}}_{k}$ for the three data sets plotted against *ω_k_*. For 43 PPS sites in rbcL and one PPS site in HA, we find that ${\bar{S}}_{k}<0$. This shows PPS sites are effectively under a mixture of purifying selection against deleterious amino acid substitutions, and diversifying selection in favor of a few amino acids that substitute rapidly among each other. This trend is evidenced when studying the pattern of PPS substitution in the influenza HA protein. The H1N1 influenza virus entered the human population sometime prior to the 1918 influenza pandemic (Taubenberger et al. 2005; dos Reis et al. 2009) and has remained largely as a single lineage since then, except from the introduction of a separate lineage of reassortant H1N1 swine virus in the 2009 pandemic (Smith et al. 2009). Figure 3 shows the pattern of amino acid substitution for the 18 PPS sites in influenza HA between 1918 and 2009. For example, site 3 remained virtually fixed for alanine between 1918 and the late 1990s, and then suffered several back and forth substitutions between alanine and valine between the late 1990s and 2009, whereas site 142 has been characterized by shifts between lysine and asparagine between 1918 and 2009. It is clear from figure 3 that the majority of PPS sites in the HA protein are characterized by back-and-forth substitutions among a fairly reduced set of amino acids.

## Discussion

Mutation–selection models of codon substitution have been successfully used to study the distribution of selection coefficients in proteins (Rodrigue et al. 2010; Tamuri et al. 2014), to detect selection shifts during adaptation (Parto and Lartillot 2017), shifting balance (Jones et al. 2016), and to understand protein evolution given structural constraints (Youssef et al. 2020). Previous works have also accommodated a *ω* parameter within the mutation–selection model to detect adaptation at amino acid sites (Yang and Nielsen 2008; Rodrigue and Lartillot 2017; Rodrigue et al. 2021). However in these works *ω* is a separate parameter and not a function of the selection coefficients and thus its population genetics interpretation is not clear (Rodrigue and Lartillot 2017). Here, we extended the mutation–selection framework to the case of PPS without the need for the additional *ω* parameter. Instead, in the new model, *ω* is a function of the selection coefficients and we believe this modeling approach can help gain insight on the nature of protein adaptation.

The new PPS model is flexible as it appears to have performed well for the different modes of selection studied here. For example, rbcL is the major subunit of the RuBisCo enzyme responsible for the fixation of carbon during photosynthesis. The efficiency of RuBisCo is affected by environmental factors and rbcL has been under persistent adaptive pressures during the successful adaptive radiation of angiosperms around the ecoregions of the world (Kapralov and Filatov 2007; Parto and Lartillot 2018). This is akin to the environment change model envisaged by Kimura and Ota (1970). On the other hand, the influenza HA protein is the classic example of positive selection on a pathogen evading its hosts’ herd immunity (Fitch et al. 1997), and we showed here the PPS model performed well in detecting this mode of adaptation. We believe the new PPS model, together with previous mutation–selection models that relaxed the assumption of constant fitnesses (Tamuri et al. 2012; Parto and Lartillot 2017), now encompass the major modes of selection in proteins.

We would like to note here two features of coding-sequence evolution that are ignored in our formulation of the PPS mutation–selection model. First, the model assumes amino acid sites within the protein evolve independently. This is unrealistic because amino acids are linked and their substitution pattern is affected by interactions with other amino acids within the folded protein (Pollock et al. 2012; Youssef et al. 2020). In particular, substitutions toward suboptimal amino acids can be compensated by rapid substitution in another interacting amino acid, so as to reduce contact energies in the folded protein (Pollock et al. 2012). How these rapid substitutions affect evolutionary dynamics within PPS and how they should be accommodated within the inference model will require further research (Youssef et al. 2020). Second, the model assumes polymorphism is absent and new mutations either become fixed or lost instantaneously. This assumption appears reasonable for most populations of plants and animals because, in these, the scaled mutation rates, Nμ, are much less than one (Lynch and Conery 2003). Even for influenza, a fast-evolving RNA virus, estimates of Nμ are in the order of $10^{-3}$ (Zhao and Illingworth 2019). However, levels of standing polymorphism can be substantial in many microorganisms (Lynch and Conery 2003) or for some loci under certain forms of selection (e.g., selection in favor heterozygotes, Hughes and Nei 1988). Incorporating polymorphism within the mutation–selection inference machinery will be challenging, but recent polymorphism-aware phylogenetic approaches may provide a way forward (De Maio et al. 2015).

Perhaps the most important insight from the application of the PPS model to real data is that the criteria ω>1 to detect positive selection in proteins is conservative. As we show here, sites under PPS are also under strong purifying constraints, and, at equilibrium, produce many deleterious mutations that are removed by selection. Because *ω_k_* is the weighted average over the rate of all possible synonymous substitutions at a site, it follows *ω_k_* will be reduced if there are many deleterious mutations at the site even if the site is shifting rapidly among a few positively selection amino acids. We believe this insight should be incorporated into the much faster codon substitution models used in phylogenomic analyses, such as the branch-site model (Yang and Nielsen 2002), to improve power in detecting adaptation in proteins.

## Materials and Methods

### Maximum Penalized Likelihood Estimation and Likelihood Ratio Test of PPS

The swMutSel model (Tamuri et al. 2012, 2014) is the special case of swMutSel-PPS when *Z_k_* = 0. We use swMutSel as a null model ($H_{0}:Z_{k}=0$) and swMutSel-PPS as the alternative model ($H_{1}:Z_{k}>0$) in a likelihood-ratio test. The vector of fitnesses at site *k*, $F_{k}=(F_{i,k})$ and the PPS component, *Z_k_* are estimated by maximizing a penalized likelihood. The penalty on $F_{k}$ is the Dirichlet-based penalty of Tamuri et al. (2014), whereas for *Z_k_* we use an exponential penalty $P(Z_{k})=e^{-\lambda Z_{k}}$, where the regularization parameter, *λ*, controls the strength of the penalty. When *λ* = 0, there is no penalty while λ>0 leads to increasingly stronger penalties on the estimation of *Z_k_*. During inference, the $q_{ij,k}$ (eq. 2) are scaled in terms of the expected number of neutral substitutions per site (Tamuri et al. 2012). This guarantees all sites are normalized to the same timescale. To speed up computation, the mutational parameters, required to construct *μ_ij_*, and the branch lengths on the phylogeny are estimated under the FMutSel0 model (Yang and Nielsen 2008) as explained in Tamuri et al. (2014). We note only the differences among the $F_{i,k}$ enter equation (2), thus, the fitness for the most common amino acid at site *k* is set to zero. Large negative $F_{i,k}$ values are capped to −10 during numerical optimization. We recommend the optimization routine is repeated three times using different parameter start values to ensure convergence to the correct estimates.

Let the maximum penalized log-likelihood for site *k* be $\ell_{0,k}$ and $\ell_{1,k}$, under the *H*_0_ and *H*_1_ hypotheses respectively. The test statistic is the difference in log-likelihoods $\delta_{k}=\ell_{1,k}-\ell_{0,k}$. If the test statistic is significantly different from zero, this is evidence site *k* is evolving under PPS. The distribution of the $2\delta_{k}$ statistic, when the null hypothesis is true, does not follow a $\chi^{2}$ distribution. There are two reasons for this. First, because *Z_k_* = 0 is at the boundary of parameter space, the test statistic would be, asymptotically, distributed as a 50:50 mixture of a $\chi^{2}$ distribution and a 0.5 point probability mass at 0 (Self and Liang 1987; Goldman and Whelan 2000). The second reason is that the penalty on *Z_k_* affects the 50:50 proportion because the penalty forces the estimates of *Z_k_* toward zero.

Because we do not know what the asymptotic distribution of *δ_k_* should be, we use Cox’s (1962) Monte Carlo simulation to obtain the null distribution of *δ_k_*. For a given site *k* in the alignment, we simulate *N* replicate sites on the phylogeny using the maximum penalized likelihood estimates (MPLEs) of $F_{k}$ under *H*_0_. The distribution, Δ_*k*_, is determined by the difference in log-likelihood between the two models for each simulated site: $\Delta_{k}=(\delta_{k}^{\left(1\right)},\delta_{k}^{\left(2\right)},\ldots ,\delta_{k}^{\left(N\right)})$ where $\delta_{k}^{\left(i\right)}=\ell_{1,k}^{\left(i\right)}-\ell_{0,k}^{\left(i\right)}$ is the log-likelihood difference for the *i*-th simulation. If the test statistic from the real data (*δ_k_*) is larger than, say, 95% of Δ_*k*_, we reject the null hypothesis *H*_0_ (no PPS) and accept the alternative hypothesis *H*_1_ (PPS) at the α=0.05 significance level. Cox’s approach has been shown to work well in phylogenetic data sets (Goldman 1993). When analyzing an ensemble of sites in a multiple sequence alignment, we correct for multiple testing using a FDR procedure to select candidate PPS sites (Benjamini and Hochberg 1995).

### Padé Approximation to Calculate the Matrix Exponential

Calculation of the likelihood along a branch of length *t* in the phylogeny requires calculation of $P_{k}(t)=\text{exp}\;tQ_{k}$, where $Q_{k}=(q_{ij,k})$ is the substitution matrix (eq. 2). However, because the PPS model is irreversible, the usual Eigen decomposition algorithm used to calculate $P_{k}(t)$ is not stable (Yang 2014). Here, we use the Padé approximation (Moler and Van Loan 2003):

$$\text{exp}\;A\approx R_{qq}(A)={\left[D_{qq}(A)\right]}^{-1}N_{qq}(A),$$

where $N_{qq}(A)=\sum_{i=0}^{q}c_{q}(i)A^{i},\;D_{qq}(A)=\sum_{i=0}^{q}c_{q}(i){\left(-1\right)}^{i}A^{i}$, and $c_{q}(i)=(2q-i)!q!/(2q)!i!(q-i)!$. Note $\text{exp}\;A={\left(\text{exp}\;A/m\right)}^{m}$, with $m=2^{j}$ for some integer *j*. Accuracy is improved considerably by choosing a suitable *j* such that the Padé approximation works well for exp A/m. Then,

$$\text{exp}\;A\approx {\left[R_{qq}(A/2^{j})\right]}^{2^{j}}.$$

Appropriate values for *q* and *j* are chosen according to the size of **A** and the desired accuracy in the calculation of exp A (Moler and Van Loan 2003: table 1).

In our model, the calculation of the likelihood at a site involves multiple computations of exp tQ for every branch in the phylogeny. We choose *q* and *j* according to the largest branch length *t*. Because ${\left(tQ/m\right)}^{i}={\left(t/m\right)}^{i}Q^{i}$, we calculate all necessary $c_{q}(i)$ and $Q^{i}$ once and cache these in memory throughout the likelihood calculation. Calculating $Q^{i}$ once is more efficient than setting A=Qt and applying the Padé approximation directly. Instead, we compute $B^{i}={\left(t/m\right)}^{i}Q^{i},\;R_{qq}(B)$, and finally ${\left[R_{qq}(B)\right]}^{2^{j}}$ for each value of *t*. We found this matrix exponentiation algorithm is approximately 1.5 times faster than the Taylor series approximation suggested in phylogenetics (Yang 2014), albeit using more memory to store the precalculated matrix powers.

### Simulated Data

To test the specificity and sensitivity of the LRT for PPS, we simulated sites on a balanced 512-taxa tree with branch lengths equal to 0.0125 neutral substitutions per site (Tamuri et al. 2014). We simulated sites under a null model with no PPS ($H_{0}:Z_{k}=0$), and under the alternative model with PPS ($H_{1}:Z_{k}>0$) with three strengths of selection $Z_{k}=\{2,5,10\}$, and with 1,000 sites simulated under each model setup. Following Tamuri et al. (2014), amino acid fitnesses for each site were sampled from a bimodal normal distribution with ten randomly selected amino acids chosen to have F∼N(0,1) and the remaining amino acids to have F∼N(−10,1). This simulation setup was chosen because it leads to simulated data that captures two important features seen in real data: 1) A bimodal distribution of selection coefficients among mutations, and 2) a sharp distribution of amino acid preferences among sites (Tamuri et al. 2012, 2014).

Simulated data were then analyzed with the swMutSel software to estimate model parameters. The branch lengths and mutation parameters were fixed to their true values (*k* = 2, $\pi^{*}=0.25$) throughout the analysis and only the sitewise fitnesses $\left(F_{k}\right)$ and diversifying selection $\left(Z_{k}\right)$ parameters were estimated. For each simulation setup, we calculated the MPLE and the LRT as described above, using *N* = 100 replicates in Cox’s procedure. In all analyses, the Dirichlet penalty on $F_{k}$ has α=0.01, and three strengths of penalty on *Z_k_* were tested, λ={0.01, 0.5, 1.0}.

Using the LRT results, we determined the false-positive and false-negative rates. The false-positive rate is calculated by determining the number of tests that incorrectly rejected the null hypothesis (*Z_k_* = 0). The true-positive rate is calculated from the number of tests that correctly rejected the null hypothesis ($Z_{k}>0$).

### Real Sequence Data

We downloaded 3,120 HA protein-coding sequences of human influenza H1 viruses (excluding 2009 pandemic-H1N1 and partial sequences) from the NIAID Influenza Research Database (Squires et al. 2012); we downloaded 3,490 RuBisCO eudicotyledon sequences from a previous study (Stamatakis et al. 2010); and we downloaded CYTB genes of placental mammals from NCBI RefSeq (O’Leary et al. 2016) mitochondria genomes. We reduced the HA and RuBisCO data sets to 466 and 478 sequences respectively by using CD-HIT (Fu et al. 2012) with clustering thresholds of 99.3% and 96% of amino acid sequence identity. The CYTB data were reduced to 418 sequences by keeping one sequence per mammal genus. Sequences were aligned using PRANK (Loytynoja and Goldman 2005), and the alignments used to estimate tree topologies with RAxML under the GTRCAT model (Stamatakis 2014). Because the swMutSel-PPS model is irreversible, trees must be rooted. Thus, outgroups were used to root the trees: avian influenza (HA), monocotyledons (RuBisCO), and monotremes (CYTB). Outgroups were removed and analyses carried out on the rooted ingroup tree (for the PPS model), and the unrooted ingroup tree (for the no PPS model). Sites with residues in fewer than 50 taxa were not analyzed. This corresponds to 31, 23, and 27 sites in the rbcL, HA, and CYTB alignments respectively. We note *Z_k_* is not identifiable if a site is conserved for a single amino acid. Such conserved sites have the same likelihood under the *H*_0_ and *H*_1_ hypotheses. MPLE and LRT were carried out as described above using α=0.01 in the Dirichlet penalty. We note estimates of $F_{i,k}$ are different between the two models (*Z_k_* = 0 vs. *Z_k_* > 0, supplementary fig. S1, Supplementary Material online). Before carrying out the FDR correction to select candidate sites under PPS, we verify the distribution of *P* values is uniform (supplementary fig. S2, Supplementary Material online).

## Supplementary Material

Supplementary data are available at *Molecular Biology and Evolution* online.

## Supplementary Material

msab309_Supplementary_Data
