# Supplementary material for: A Mutation–Selection Model of Protein Evolution under Persistent Positive Selection
Source: Mol Biol Evol. 2021 Oct 25;39(1):msab309. doi: 10.1093/molbev/msab309 (PMC8760937; doi:10.1093/molbev/msab309)
Supplement: msab309_Supplementary_Data [file msab309_supplementary_data.zip › SupplementaryInformation-20211014.pdf]

Supplementary Information for "A mutation-selection model of protein evolution under persistent positive selection"

By Asif Tamuri and Mario dos Reis

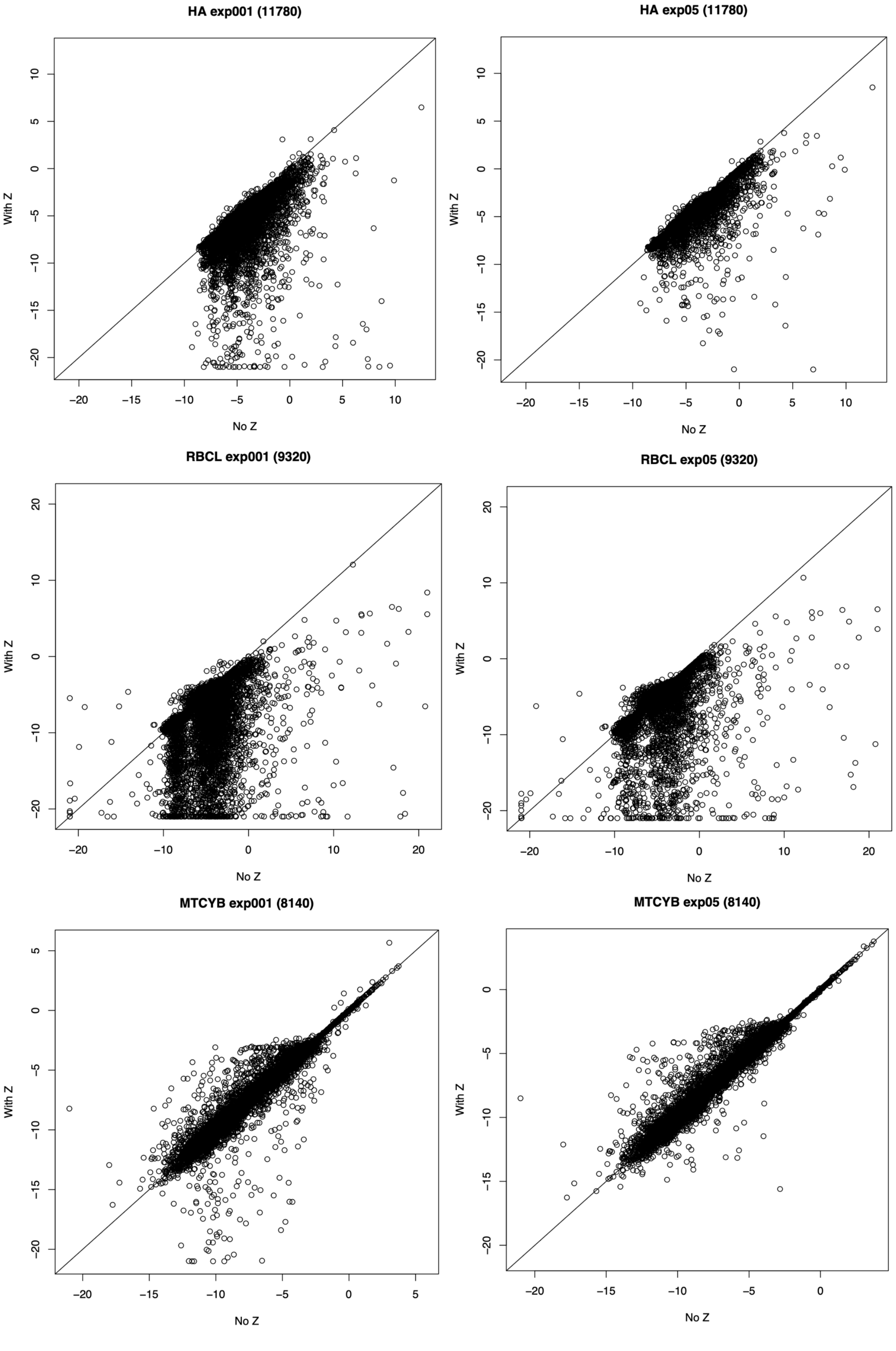

Figure S1. Estimates of $F_{i,k}$ when $Z_k = 0$ (x-axis) are plotted against the $F_{i,k}$ estimates when Z_k is estimated (y-axis) for all three real datasets and under different exponential penalties ($\lambda = 0.01, 0.5$). The number of $F_{i,k}$ values estimated in each dataset is shown in brackets.

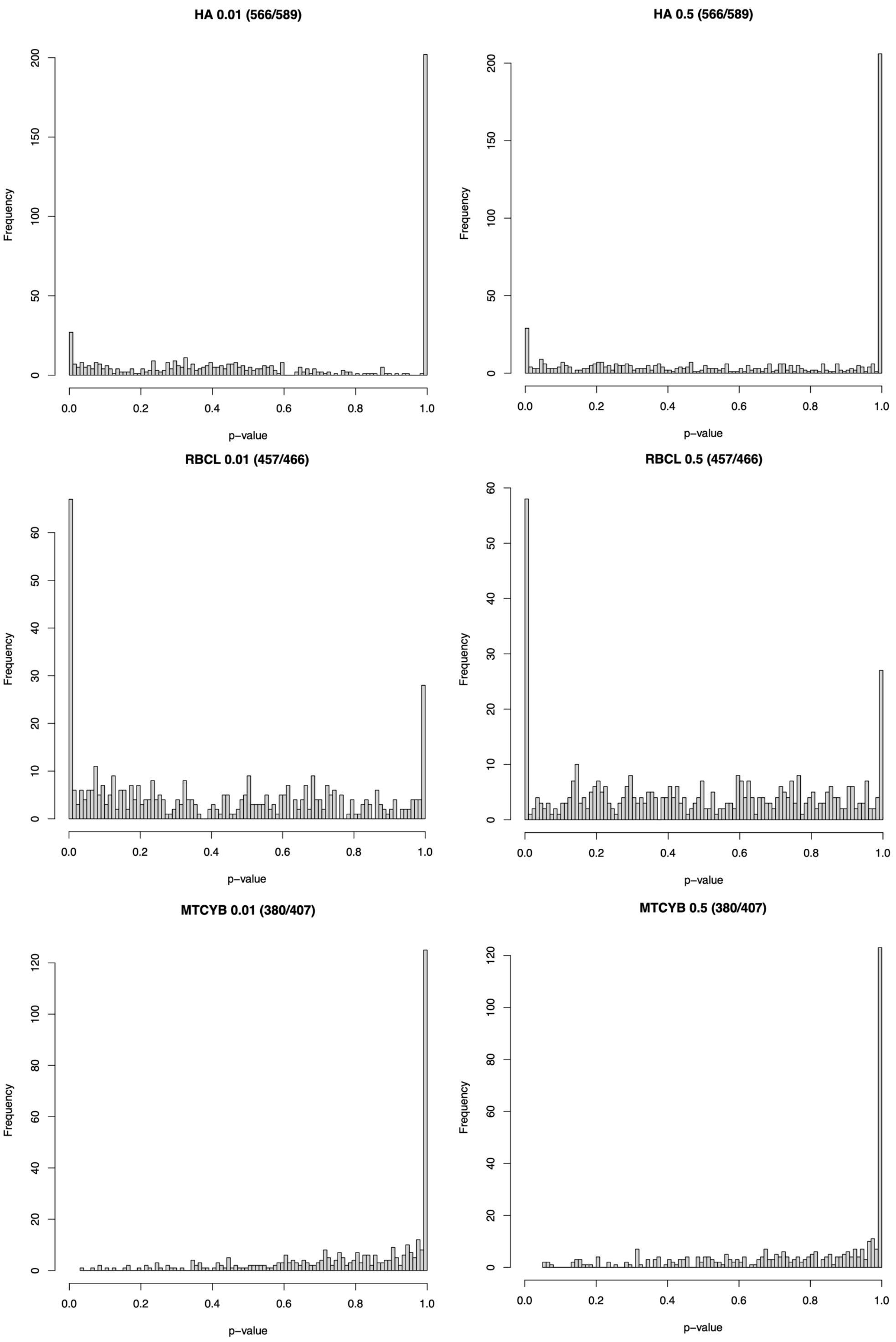

Figure S2. Distribution of p-values for the LRT of the alternative hypothesis H_1: $Z_k > 0$ (PPS) against the null hypothesis H_0: $Z_k = 0$ (no PPS) for the three real datasets under different exponential penalties (λ = 0.01, 0.5). The p-values are obtained using Cox simulation approach. The peak of p-values at 1 is due to the test being one sided (e.g., $Z_k > 0$). These values are removed before application of the false discovery rate procedure.
